# A qualitative study exploring the influence of a talent management initiative on registered nurses' retention intentions

**DOI:** 10.1111/jonm.13885

**Published:** 2022-11-12

**Authors:** Nicola Fisher, Louise Bramley, Joanne Cooper, Sarah Elizabeth Field‐Richards, Joanne Lymn, Stephen Timmons

**Affiliations:** ^1^ Nottingham University Business School University of Nottingham Nottingham UK; ^2^ Institute of Care Excellence Nottingham University Hospitals NHS Trust Nottingham UK; ^3^ School of Health Sciences University of Nottingham Nottingham UK

**Keywords:** human resource management, networks, nurses, retention, social capital, talent management

## Abstract

**Aim:**

The aim of this study is to explore the influence of a talent management scheme in an English National Health Service (NHS) Trust on registered nurses' retention intentions.

**Background:**

The retention of nurses is a global challenge, and talent management initiatives can play a role in improving retention. Talent management in its broadest sense is a way in which an organization recruits and retains the workforce that it needs to optimize the services it delivers.

**Methods:**

In this qualitative study, eight in‐depth semi‐structured interviews were conducted with registered nurses who had participated in a talent management initiative, at an English acute NHS Trust. Data were collected in July 2019.

**Results:**

The talent management initiative influenced positive retention intentions. Retention of nurses was facilitated by the creation of networks and networking.

**Conclusion:**

Networks and networking can be viewed as a form of social capital, which was a facilitating factor for positive retention intentions for nurses.

**Implications for Nursing Management:**

Talent management initiatives for nurses should be developed and directed to include the building of networks and networking to enable development of social capital. Although this talent management scheme is within the NHS, the issue of nursing retention is global. Application of learning from this paper to other health care systems is possible.

## 1 INTRODUCTION AND BACKGROUND

The retention of nurses in the workforce is recognized as a global challenge and is central to ensuring that health services are accessible and of good quality. Low retention rates are a concern as nurses are the largest safety‐critical profession and demand for nurses is only expected to grow (Halter et al., 2017). The underlying causes of nursing shortages are multi‐faceted, and as a result, solutions to retention are complex (Buchan & Aiken, 2008).

In the NHS, there is currently a shortage of nurses, with a 10.5% vacancy rate in England (NHS Digital, 2021). While recruitment has often dominated the discourse around this shortage, retention of staff is gaining significant attention (Buchan et al., 2019; Nuffield Trust, 2022). Reviews have indicated that a lack of career development are a reason for nurses leaving (House of Commons Health Committee, 2018). Therefore, this places talent management as a key component in helping to alleviate nurse retention issues. In this article, we debate the key concepts of talent management and report on a study that explores the impact of a talent management programme on nurses' retention intentions.

### 1.1 Nursing retention

Predictive elements that have been shown to improve nurse retention include autonomy, empowerment, job satisfaction, career development and organizational practices including managerial style, placement opportunities and supervisory support (Mills et al., 2016). As a result, health care organizations are formulating talent management initiatives, which can enable them to retain their experienced and skilled workforce. Talent management can impact upon organizational performance, including retention (Dahshan et al., 2018). However, few talent management schemes have been evaluated for their specific benefits (Haines, 2016).

### 1.2 Talent management theory and concepts

**FIGURE 1:**
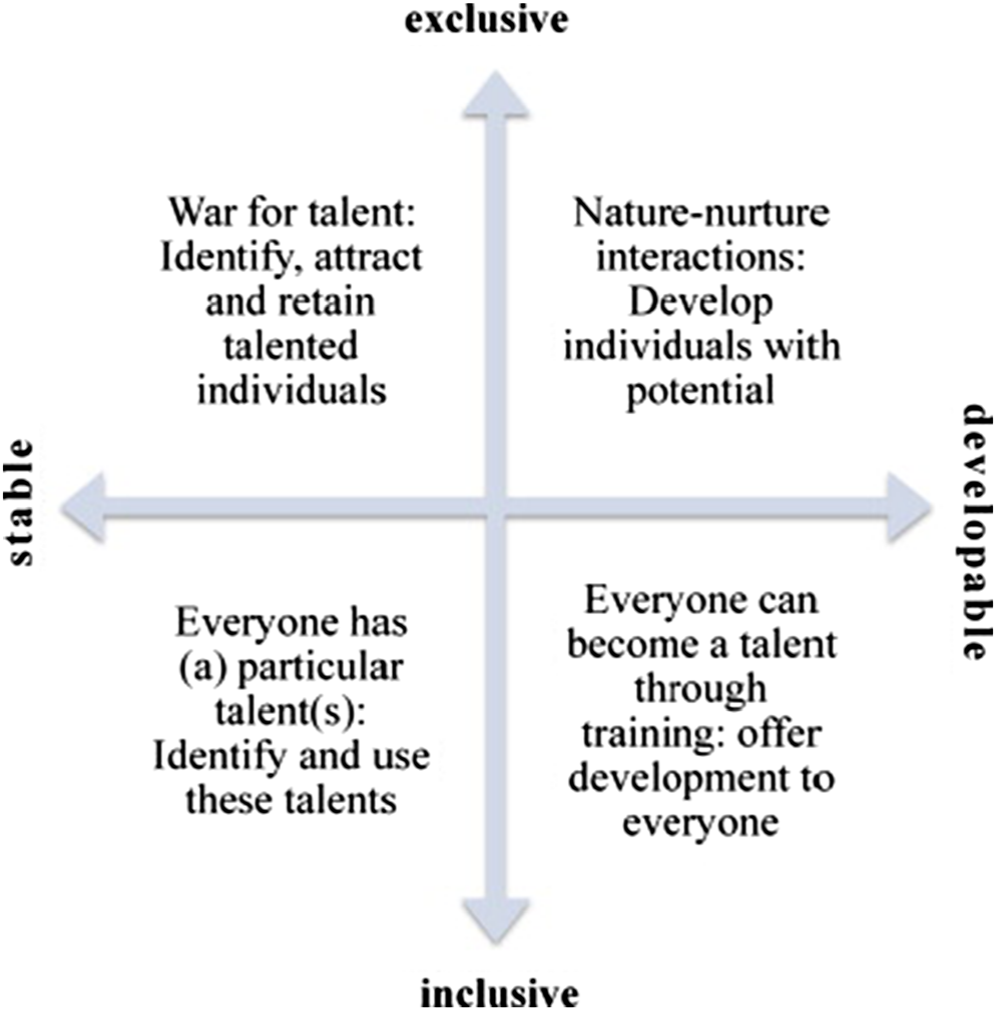
Philosophies of ‘talent’ management. Meyers and von Woerkom (2014a)

Talent management involves ‘growth from within’ and incorporates the development of employees and not just their utilization for organizational benefit. It is thus imperative organizations develop the ‘human capital’ of their employees. Human capital encompasses the knowledge and ideas of individuals, which includes their emotional intelligence, values, motivations and inter‐personal skills (Bottone & Sena, 2011). Social capital, defined here as the value of connections, and knowledge and resources embedded within networks of relationships (Nahapiet & Ghoshal, 1998; Youndt & Snell, 2004). It is an emerging field in nursing research (Read, 2014), which could help create and develop human capital (Coleman, 1988). Nurses' social capital has positive consequences for nurses, their patients and health care organizations (Xu et al., 2020). Indeed, social capital could support talent management by influencing nurse retention, including a greater sense of job satisfaction, which is indicated in enhancing nurses' retention (Read & Laschinger, 2015). Talent management in its broadest sense can be considered as a way in which an organization constructs and renews the type of workforce that it needs to be successful. There is no single definition of talent, but it can be viewed through four primary lenses (Figure 1).

Variations in the definition of talent impact upon its management. An inclusive, developable lens considers that all employees can grow and change and has an underlying assumption that individuals have an intrinsic need to fulfil themselves (Meyers & von Woerkom, 2014b). Proponents of an inclusive, developmental perspective state that this approach also enhances wellbeing through positive psychology and considerations of the Capability Approach, with benefits for the individual and organization (Swailes et al., 2014). This study views talent management from an inclusive perspective for reasons we will explain below.

### 1.3 Talent management in health care and nursing

Talent management within the NHS has moved to become more inclusive and developmental due to criticism for not being able to meet the dynamic and complex demands of service (NHS Leadership Academy, 2015). One challenge, however, is understanding how talent management schemes are, or are not, successful in helping to retain nurses. The concept of talent management within nursing is not well established, despite individual components of talent management such as retention and development gaining attention.

### 1.4 The chief nurse excellence in care junior fellowship

One example of a talent management initiative, the Chief Nurse Excellence in Care Junior Fellowship (CNF), was developed in a large acute NHS Trust in 2016. NHS Trusts are public sector bodies that provide health care for a particular geographical area and are normally either acute, community, ambulance or mental health service focused. The term junior was used to identify the fellowship as an early career opportunity. It is part of a wider fellowship development programme that also incorporates opportunities for mid and late career nurses.

The CNF programme was developed in response to challenges in the retention of nurses and feedback indicating staff wanted more development opportunities. The CNF aims to develop participants' skills in change management, innovation, improvement science and leadership. The target group were Agenda for Change (AfC) band 5 nurses, as they make up a large proportion of the nursing workforce (53%) in the Trust. AfC is the national pay scale for nurses and other health care professionals in the NHS, and band 5 is the level at which nurses enter as registered staff. While this programme was initially aimed at nurses, it has subsequently expanded to AHPs and Midwives and nurses on other AfC bands.

The CNF takes place over 12 months with 1 day per week out of clinical practice and has two components. First is a bespoke development programme, underpinned by an inclusive talent management approach. The programme includes one‐to‐one mentorship and opportunities for public and patient engagement. Depending on prior knowledge and experience, there is also the provision of specific training including quality improvement methodology and project financial management. Second is a quality improvement project that focuses on an area pertinent to clinical practice and is aligned to organizational and nursing‐specific strategic objectives (Bramley et al., 2018).

Mentors from either nursing, midwifery or AHP backgrounds who have clinical academic expertise (many of whom are post‐doctoral) work collaboratively with clinical leaders, for example, specialist nurses, advanced clinical practitioners and ward leaders, to develop the individual knowledge and skills and projects of the CNFs. Previous projects have included enhancing dementia care in recovery (Edis, 2017) and enhancing the care of patients' neutropenic sepsis patients (Leighton, 2018).

In order to understand the impact of the CNF fellowship as a talent management programme on the retention intentions of staff, further research was warranted. The lead author of this paper, who is external to the Trust and has a specialist interest in nurse retention, conducted the research and analysis independently from the programme leaders. The aim of this study was to explore the impact of the CNF initiative on retention intentions of staff.

## 2 METHODS

### 2.1 Sampling/participants

Participants were recruited with the help of gatekeepers within the Trust. Between 2016 and the time of the research (July 2019), 23 individuals had been CNFs with 21 remaining in the organization. An email sent out to the current and alumni CNFs with a participant information sheet. The sampling approach was purposive due to the specific targeting of those who had or were undertaking the CNFs scheme, enhancing the trustworthiness of the study outcomes (Elo et al., 2014). Participants who were interested contacted the researcher directly. Out of a possible 21 respondents, eight responses were received, and eight interviews were conducted. Seven AfC band 5s, one AfC band 6, whom had between 2 and 6 years of clinical experience post‐qualification were interviewed. Of these, seven were registered nurses (six adult nurses, one children's nurse, of these seven, six were female, and one was male), and one was an Operating Department Practitioner (female).

### 2.2 Data collection

Semi‐structured interviews were utilized to gain an in‐depth understanding of the scheme from the participants' perspectives. Interviews were conducted either face to face (taking place in participants' Trust work areas, in a Trust corporate office or within a University room) or via telephone. A choice of interview type and location was provided to participants to maximize flexibility and convenience for participants and aid in recruitment. All interview locations were in private areas, to minimize noise, distractions and interruptions and protect confidentiality. An interview schedule developed with Trust partners was used in both face‐to‐face and telephone interviews. Questions exploded aspects of participants' development that had benefitted from the CNF scheme, previously identified from survey data collected by the Trust, and how participants felt this had occurred, focusing on the influence of these on their retention outlook. The interview schedule was reviewed by the study team (ST, JL and SFR) prior to the interviews to ensure clarity and appropriateness of questions. All interviews were conducted by the lead author of this paper and lasted for an average of 45 min (with average length of face‐to‐face and telephone interviews 40 min and 50 min, respectively). Both face‐to‐face and telephone interviews were audio recorded using a voice recorder.

### 2.3 Data analysis

Interviews were transcribed verbatim and then read through in full to check for accuracy alongside the audio recordings. The transcripts were analysed thematically following Braun and Clarke (2006) using an inductive approach. The inductive approach derives analysis from the raw data, not from pre‐existing theoretical models, which enabled the analysis to remain open to all possible influences on retention and the outcomes from the CNF. Codes, themes and developing interpretations were discussed in the study team, to promote criticality, rigour, transparency and credibility. The main consideration for analysis was in relation to the CNF's impact on participants' retention intentions. The study team then looked for factors which impacted these intentions, which subsequently formed the further themes: career development and networks and networking.

### 2.4 Ethical considerations

This evaluation corresponds to the definition of a service evaluation according to the Health Research Authority (HRA) (2017), which was supported by the Trust, meaning it does not require NHS Research Ethics Committee approval. A service evaluation considers how well a service is achieving its intended aims and is conducted to judge the merit of that service. However, full ethical principles were still followed. Participants were informed that any data generated from the interviews, and their confidentiality would be protected under the *Data Protection Act 1998* and the *European Union General Data Protection Regulation*. All names of the participants and those that they mentioned in their interviews were anonymized. Participants were given a consent form to sign and had the right to withdraw at any time. There were no risks identified to participants.

## 3 RESULTS

### 3.1 Retention intentions

Participants expressed positive views in relation to the CNF, which they felt impacted their retention intentions. These included feeling valued, invested in, and that their ideas and opinions were listened to. Participants also expressed that this brought with it a sense of feeling connected, while still being recognized as an individual and less like a “small cog in a big wheel” (Participant 6). This meant that they felt more positive about staying at the Trust.

Participant 1 … I feel more valued, I feel like my opinions and ideas are important because I didn't feel like that before … there is more of a connection to the Trust now as a whole.

Participants spoke about how having the exposure to the leadership of senior nurses enabled them to see how motivated the Trust was in terms of retention and encouraging development. Some participants felt that this brought with it a sense of loyalty towards the Trust, while others felt that due to this exposure, they were able to feel less like a “worker ant” (Participant 6).

Participant 2 … I feel if you feel invested in, then I want to stay somewhere because then I feel a sense of loyalty back to them … I feel it's so important to know that someone cares about how I develop as an individual.

Participant 4 … made me feel like this is a really good place to work and this is the kind of people I want to learn from and this is the nursing leadership I want to be under … they've given me this opportunity so now I don't have a reason to leave.

Finally, it was indicated that the CNF supported retention in the profession, even if not to the Trust. From the CNF, participants could see what they wanted and needed, and it (the CNF) allowed them to apply elsewhere for a job that met those needs rather than them staying in the same job and getting disillusioned potentially resulting in loss from the profession.

Participant 8 … I was not given or were not able to access the same opportunities after the Chief Nurse Fellows scheme in the Trust so I looked elsewhere.

### 3.2 Career development

Career development was noted to be a significant factor for participants in relation to their positive retention intentions. The majority of participants said that the skills and development they had gained from the CNF had resulted in them getting new jobs, whether that be a promotion, or enabling them to put a “foot in the door” (Participant 5).

Participant 3 … I was looking at band 6s and what criteria was needed and then I saw that [CNF scheme advertised], and thought that will be really good, because at that point I didn't feel quite ready to apply for a band 6. It [CNF scheme] is really good for transition.

Participants also spoke about being able to step back and see what other opportunities there were, which took them out of the “silos of nursing” (Participant 2). Participants spoke about the importance of movement and being able to “try on” (Participant 4) different components that helped them to gain an insight into different career areas.

Participant 5 I like change, and if I felt like I was stuck in an area with nowhere to go I think that would not be good for me. That would really put me off working somewhere.

All participants spoke about how important their career development was to them, including reasons such as the desire to improve themselves and patient care. They felt that if there were no opportunities, they would look elsewhere. This intersection between career development and retention were discussed regularly. However, the opportunities that came from the exposure due to the CNF scheme needed to be capitalized on after it had finished.

### 3.3 Networks and networking

A facilitating factor for retention and career development was networks. This came in two forms. First, the building of networks was considered by participants to aid their personal and professional development. For example, participants felt that networks acted as support mechanisms for personal and professional wellbeing, which were reflected through the mentorship elements, and being within a cohort of other CNFs.

Participant 1 the mentor has been really supportive which has helped me put myself out there and get noticed by senior nurses and consultants…feel that it gives you a spotlight to say look I am here, I can do these things and then you get more opportunities.

Additionally, participants commented that through attending conferences and meeting people with similar areas of interest to them, visiting other Trusts to see their practices and joining clinical groups such as the Long‐Term Conditions Group, helped them to develop their working practices and working connections.

Participant 4 the networking helped to push me out my comfort zone massively … It's been big for my personal development.

It was noted that participants did not feel that the opportunities for these developments would have been available had they not had the exposure to the networking as part of the scheme. Furthermore, participants commented that it was also the exposure to people within different career areas that enhanced their ability to explore their own development. Being able to make connections with people to ask questions and gain guidance appeared to be a facilitating factor in career development for participants. This was emphasized by participants as being important because there were no national pathways for career development. For instance, an example was given that within the Emergency Department, a more “natural progression” (Participant 2) was to advanced clinical practice and that for participants who did not want to go into that, it was harder to explore other options unless there were role models in their clinical area.

The second factor was a combination of understanding the broader picture within the Trust and feeling part of a network. Participants commented that the exposure through networking made them realize why things were happening and that “there was a reason for all of this” (Participant 4) and the effort that the Trust was putting in to making things better for staff. Participants commented that this made them feel more connected and “plugged in” (Participant 5), that it allowed them to realize that they had more influencing power and that made them feel part “something bigger” (Participant 7). In some cases, it cultivated a stronger sense of wanting to stay in the Trust as a result.

Participant 2 … if you don't have those embedded connections and people that you can go to and that network that exists, I feel like you just flounder…

Finally, participants felt that they gained a greater sense of empowerment through the interconnected impact of the CNF scheme, which helped to dispel some of the ingrained hierarchy structures within the organization. This helped participants to feel that they gained greater confidence to challenge and raise concerns.

Participants 1 It felt less like [referring to the clinical nursing directors] that was them up there, and this was me down here

Participant 6 [the CNF] helped me to realise the influencing powers nurses have on a day‐to‐day basis

## 4 DISCUSSION

The findings from this study suggested that the CNF had a positive influence upon personal and professional development, and the retention of participants was noted to be mediated by the presence of career development. This came in the form of participants being able to gain an awareness and opportunity to explore different career options. Furthermore, the creation of networks and networking through the CNF appear to have been an influential facilitating aspect from the view of participants in developing them professionally and personally, while enhancing their career development and retention prospects. Additionally, although the CNF cannot be solely responsible for retention, it appeared to have a positive effect on participants' views of the Trust and the ability to enhance their development. Overall, the CNF appear to have positively influenced the retention intentions of participants. Since this study was undertaken, the COVID‐19 pandemic has had a significant impact on nurse turnover and retention, with research highlighting stress and burnout as a significant factor (Falatah, 2021). Just prior to the pandemic, the 2020 cohort of 24 CNFs were recruited, and further research is required to understand this programme in a post‐pandemic system.

As social capital is considered to be the connections, knowledge and resources embedded within networks of relationships, the benefits of network and networking that participants expressed can be considered social capital and appeared to be a core part of facilitating positive retention intentions of nurses from the CNF. As social capital is noted in this study to be a key factor, it could be considered by nurse managers in the development of talent management schemes to help contribute to their organization's retention strategy. In this discussion, the facilitating presence of social capital will be considered in relation to retention.

Although career development and progression were recognized to be individual decisions pivotal to retention intentions, it was the presence of networks and networking in this study which contributed to the increase in awareness of careers, and, in turn, increased the feelings of loyalty and value expressed by participants. This sense of job satisfaction resulting from social capital has previously been shown to lead to positive retention outcomes in nursing (Read & Laschinger, 2015), and nurses' workforce social capital is indicated to hold positive consequences for nurses, their patients and health care organizations (Xu et al., 2020). At the time of writing there is no literature available linking social capital, talent management and retention in nursing, making this paper the first one to do so. However, there is research from fields outside of nursing that indicate that social capital and talent management are interconnected (Walker, 2020).

Building on the facilitating nature of social capital, research suggests that NHS Trusts could enable high‐quality patient care and safety through empowering the nursing workforce and enhancing their wellbeing (Ellis & Gates, 2005). An aspect of an empowering environment that enables this is the flow of knowledge among nurses, leadership, and other multi‐professional teams. Additionally, structural conditions such as access to information about the organization and the ability to achieve professional development at work contribute to nurses feeling empowered (Linnen & Rowley, 2014). The CNF scheme enabled exposure to information on the organization and helped career developed for participants, for example by exposing participants to leadership and organizational decision‐making processes, strategy development and various career opportunities available.

The facilitating presence of social capital relates to the importance of structural empowerment, which is a core component of retention (George, 2015; Read & Laschinger, 2015). Structural empowerment focuses on the organizational structure and personnel polices from human resource management, including talent management (George, 2015), which nurse managers can contribute to. It focuses on employees being involved in decision‐making to address areas for improvement. The concept of structural empowerment also attends to the influence leaders can have on professional practice by creating an environment that supports collaboration, which is indicated in positive retention outcomes for nurses (Halter et al., 2017). This collaborative requirement is what the CNF produces through the facilitation of networking and the creation of networks within the organization.

As an NHS Trust is a knowledge‐intensive organization, nurse managers should be aware that a key part for its success is the ability for knowledge to be transferred and shared. This requires social capital because human capital is not owned by organizations (Nahapiet & Ghoshal, 1998). Therefore, as individuals can leave, and take their human capital with them, organizations may incur a ‘capital loss’ because the knowledge may not be transferred or shared (Youndt & Snell, 2004), but social capital can help to distribute knowledge. Critically, the presence and development of tacit knowledge is important for facilitating these networks because tacit knowledge involves interpersonal skills, such as communication, which is vital to the sharing and transfer of knowledge. In this study, the CNF develops tacit knowledge including the development of presentation and teaching skills. These types of skills are vital to the development and transfer of knowledge; thus, helping to build social capital, and therefore the CNF scheme can help to reduce the risk of capital loss to nurse managers and the organization through positively enhancing retention intentions.

However, nurse managers should also be aware that there are barriers that can make the transfer and sharing of knowledge difficult. According to Youndt and Snell (2004) and Bratianu (2017), these include hierarchical and horizontal barriers, and strategies to alleviate these barriers include fostering egalitarian and collaborative human resource management configurations. This study indicates that incorporating social capital in talent management initiatives may help to alleviate these barriers. An egalitarian approach considers the need to reduce power distances within an organization through, for example, empowering individuals. Within this study, participants reported that undertaking the scheme made them feel more empowered and realize the impact nurses can have within an organization. These benefits further relate to the concept of the professional voice and wellbeing.

In terms of overcoming horizontal barriers to knowledge sharing and transfer, Youndt and Snell (2004) suggest collaborative human resource management practices, which can be achieved through work structures that promote network intimacy. The CNF scheme facilitates this, as participants reported that they had a greater understanding of how and why the Trust operated, which contributed to them having a greater appreciation for the Trust and the leaders that were trying to retain them. This subsequently made participants feel greater commitment towards the Trust, which subsequently made retention intentions more positive. The contribution of networking, and networking within talent management initiatives and retention, has not been extensively researched. Therefore, this study adds to the talent management literature, and the importance of talent management with network and networking for nurses' retention intensions. The difference between this and other studies is also that although talent management has been used in other sectors such as business, it is only an emerging concept in nursing, and Haines (2016) states that talent management approaches in nursing require evaluation, which is what this study presents, especially for nurses who are not senior leaders. This study demonstrates that talent management is a useful approach for early career nurses, not least as a retention intervention. Therefore, the contribution of this study is in describing a talent management approach in nursing which may have significant implications for retention of early career staff. Nurse managers when considering a talent management approach should think about including social capital with networks and networking within their initiatives to encourage positive retention intentions.

### 4.1 Limitations

This study has been carried out on a small sample of nurses. While the outcomes positively highlight the impact of this talent management programme for the individuals interviewed, these are focused on an acute trust only and may differ across other health care settings. Moreover, this scheme has so far only been undertaken by ‘junior’ grades, and thus, there may be differences of outcomes if ‘senior’ grade nurses undertook the CNF.

## 5 CONCLUSION

This paper has presented how a talent management scheme may influence nurses' retention intentions. The findings suggest that participants gained a range of professional and personal development because of the talent management initiative. The talent management scheme did have a positive effect on participant's views of the organization, which could improve retention intentions. A core influence on retention from within the talent management initiative was the presence of networks and networking. The networking can be viewed as a form of social capital, which stood out in these findings as being a facilitating factor between retention and career development.

With further research of the combination and interlinking of social capital within talent management initiatives in nursing, a greater understanding may be able to inform future developments. Exploring this approach; especially post pandemic, may assist nurse managers in developing retention strategies for nurses in a range of organizations and across different countries. Enhancing retention is one way of addressing NHS nursing shortages, which this talent management initiative could potentially contribute to.

## 6 IMPLICATIONS FOR NURSING MANAGEMENT

This paper suggests that networks and networking, considered here as social capital, may function as core facilitating aspects in helping to promote retention intentions for nurses within a talent management initiative. Thus, when creating or developing talent management schemes, nurse managers should give thought to how networks and networking are embedded within such initiatives for early career staff. Finally, there is the potential to advance the CNF scheme out to mid or late‐career nurses, and it may be possible to establish if the same focus on networks and networking are emphasized by these group of nurses. This may help nurse managers to develop a broader, and more in‐depth talent culture within their organization.

## CONFLICT OF INTEREST

One of the authors is a National Institute for Health Research (NIHR) Senior Nurse and Midwife Research Leader. The views expressed in this article are those of the author(s) and not necessarily those of the NIHR or the Department of Health and Social Care.

## ETHICS STATEMENT

This study corresponds to the definition of a service evaluation according to the Health Research Authority (2017), which was supported by the Trust. A service evaluation considers how well a service is achieving its intended aims and is conducted to judge the merits of that service. However, full ethical principles were still followed. Participants were informed that any data generated from the interviews, and their confidentiality, would be protected under the Data Protection Act 1998 and the European Union General Data Protection Regulation. All names of participants and those they mentioned were anonymised. Participants were given an information sheet, signed a consent form and had the right to withdraw at any time. No risks were identified to participants.

## Data Availability

Research data are not shared.

## References

[jonm13885-bib-0001] Bottone, G. , & Sena, V. (2011). Human capital: Theoretical and empirical insights. The American Journal of Economics and Sociology, 70, 401–423. 10.1111/j.1536-7150.2011.00781.x

[jonm13885-bib-0002] Bramley, L. , Manning, J. , & Cooper, J. (2018). Engaging and developing front‐line clinical nurses to drive care excellence: Evaluating the chief nurse excellence in care junior fellowship initiative. Journal of Research in Nursing, 23, 678–689. 10.1177/1744987118808843 34394489 PMC7932417

[jonm13885-bib-0003] Bratianu, C. (2017). The knowledge economy: The present future. Management Dynamics in the Knowledge Economy, 5, 477–479. 10.25019/MDKE/5.4.01

[jonm13885-bib-0004] Braun, V. , & Clarke, V. (2006). Using thematic analysis in psychology. Qualitative Research in Psychology, 3, 77–101. 10.1191/1478088706qp063oa

[jonm13885-bib-0005] Buchan, J. , & Aiken, L. (2008). Solving nursing shortages: A common priority. Journal of Clinical Nursing, 17, 3262–3268. 10.1111/j.1365-2702.2008.02636.x 19146584 PMC2858425

[jonm13885-bib-0035] Buchan, J. , Charlsworth, A. , Gerslick, B. , & Seccombe, I. (2019). A critical momen: NHS staffing trends, retention and attrition. The Health Foundation.

[jonm13885-bib-0006] Coleman, J. (1988). Social capital in the creation of human capital. American Journal of Sociology, 94, 95–120. 10.1086/228943

[jonm13885-bib-0007] Dahshan, M. , Keshk, L. , & Dorgham, L. (2018). Talent management and its effect on organisation performance among nurses at Shebin El‐Kom hospitals. International Journal of Nursing, 5. 10.15640/jns.v5n2a10

[jonm13885-bib-0008] Edis, H. (2017). Improving care for patients with dementia in the recovery room. British Journal of Nursing, 26(20), 1102–1108. 10.12968/bjon.2017.26.20.1102 29125364

[jonm13885-bib-0009] Ellis, B. , & Gates, J. (2005). Achieving magnet status. Nursing Administration Quarterly, 29, 241–244. 10.1097/00006216-200507000-00008 16056158

[jonm13885-bib-0010] Elo, S. , Kaariainen, M. , Kanste, O. , Polkki, T. , Utriainen, T. , & Kyngas, H. (2014). Qualitative content analysis: A focus on trustworthiness. SAGE Open, 14(1), 1–10. 10.1177/2158244014522633

[jonm13885-bib-0011] Falatah, R. (2021). The impact of the coronavirus disease (COVID‐19) pandemic on nurses' turnover intention: An integrative review. Nursing Reports, 11, 787–810. 10.3390/nursrep11040075 34968269 PMC8715458

[jonm13885-bib-0012] George, C. (2015). Retaining professional workers: What makes them stay? Employee Relations, 37(1), 102–121. 10.1108/ER-10-2013-0151

[jonm13885-bib-0013] Haines, S. (2016). Talent management in nursing. An exploratory case study of a large acute NHS trust [Thesis]. University of Nottingham.

[jonm13885-bib-0014] Halter, M. , Boiko, O. , Pelone, F. , Beighton, C. , Harria, R. , Gale, J. , Gourlay, S. , & Drennan, V. (2017). The determinants and consequences of adult nursing staff turnover: A systematic review of systematic reviews. BMC Health Services Research, 17(1), 824. 10.1186/s12913-017-2707-0 29246221 PMC5732502

[jonm13885-bib-0015] Health Research Authority . (2017) Defining research table [online]. http://www.hra-decisiontools.org.uk/research/docs/DefiningResearchTable_Oct2017-1.pdf

[jonm13885-bib-0016] House of Commons Health Committee . (2018). The nursing workforce. Second report of session 2017–19. HC 353. House of Commons.

[jonm13885-bib-0017] Leighton, S. (2018) Nurse‐led intervention in the neutropenic sepsis pathway at Nottingham University Hospitals NHS Trust. Atlas of Shared Learning [Online]. https://www.england.nhs.uk/atlas_case_study/nurse-led-intervention-in-the-neutropenic-sepsis-pathway-at-nottingham-university-hospitals-nhs-trust/

[jonm13885-bib-0018] Linnen, D. , & Rowley, A. (2014). Encouraging clinical nurse empowerment. Nursing Management, 45, 44–47. 10.1097/01.NUMA.0000442640.70829.d1 24472789

[jonm13885-bib-0020] Meyers, M. , & von Woerkom, M. (2014a). ‘Fig.1. Philosophies of ‘talent’ management’ In: The influence of underlying philosophies on talent arrangement: Theory, implications for practice, and research agenda. Journal of World Business, 49, 192–203. 10.1016/j.jwb.2013.11.003

[jonm13885-bib-0021] Meyers, M. , & von Woerkom, M. (2014b). The influence of underlying philosophies on talent arrangement: Theory, implications for practice, and research agenda. Journal of World Business, 49(2), 192–203. 10.1016/j.jwb.2013.11.003

[jonm13885-bib-0022] Mills, J. , Chamberlin‐Salaun, J. , Harrison, H. , Yates, K. , & O'Shea, A. (2016). Retaining early career registered nurses: A case study. BMC Nursing, 15, 57. 10.1186/s12912-016-0177-z 27766042 PMC5057224

[jonm13885-bib-0023] Nahapiet, J. , & Ghoshal, S. (1998). Social capital, intellectual capital and the organisational advantage. The Academy of Management Review, 23(2), 242–266. 10.2307/259373

[jonm13885-bib-0024] NHS Digital . (2021) NHS Vacancy Statistics England April 2015–September 2021 experimental statistics [online] https://digital.nhs.uk/data-and-information/publications/statistical/nhs-vacancies-survey/april-2015---september-2021-experimental-statistics#highlights

[jonm13885-bib-0025] NHS Leadership Academy . (2015). Talent and talent management insights. Insight 1: Defining talent and talent management. NHS Leadership Academy.

[jonm13885-bib-0026] Nuffield Trust . (2022). Peak leaving? A spotlight on nurse leaver rates in the UK [online]. https://www.nuffieldtrust.org.uk/resource/peak-leaving-a-spotlight-on-nurse-leaver-rates-in-the-uk

[jonm13885-bib-0027] Read, E. (2014). Workplace social capital in nurses: An evolutionary concept analysis. Journal of Advanced Nursing, 70(5), 997–1007. 10.1111/jan.12251 24103033

[jonm13885-bib-0028] Read, E. , & Laschinger, H. (2015). The influence of authentic leadership and empowerment on nurses' relational social capital, mental health and job satisfaction over the first year of practice. Journal of Advanced Nursing, 71(7), 1611–1623. 10.1111/jan.12625 25656433

[jonm13885-bib-0029] Swailes, S. , Downs, Y. , & Orr, K. (2014). Conceptualising inclusive talent management: Potential, possibilities and practicalities. Human Resource Development International, 17, 529–544. 10.1080/13678868.2014.954188

[jonm13885-bib-0031] Walker, T. (2020). Inclusive talent management in the public sector: Theory and practice. Transnational Corporations Review, 12(2), 140–148. 10.1080/19186444.2020.1741296

[jonm13885-bib-0032] Xu, J. , Kunaviktikul, W. , Akkadechanunt, T. , Nantsupawat, A. , & Stark, A. (2020). A contemporary understanding of nurses' workplace social capital: A response to the rapid changes in the nursing workforce. Journal of Nursing Management, 28(2), 247–258. 10.1111/jonm.12914 31793081 PMC7328727

[jonm13885-bib-0033] Youndt, M. , & Snell, S. (2004). Human resource configurations, intellectual capital and organisational performance. Journal of Managerial Issues, 16(2), 337–360.

